# Tree Tensor Network Simulation of Dynamical Quantum Phase Transitions in the 2D Transverse-Field Ising Model

**DOI:** 10.3390/e28050495

**Published:** 2026-04-26

**Authors:** Xiangyue Zhang, Dizhou Xie, Yongqiang Li

**Affiliations:** College of Science, National University of Defense Technology, Changsha 410073, China; zhangxiangyue23@nudt.edu.cn (X.Z.);

**Keywords:** dynamical quantum phase transitions, tree tensor network, 2D transverse-field Ising model, non-equilibrium dynamics, Loschmidt echo

## Abstract

The discovery of dynamical quantum phase transitions (DQPTs) has fundamentally challenged the traditional view that phase transitions only occur in thermal equilibrium. Experimental platforms and 1D numerical methods, like matrix product states (MPS), have made great progress. However, exploring true 2D DQPTs remains difficult due to finite-size limitations and the geometric biases of quasi-1D cylinder mappings. Here, we bypass these limitations by deploying a tree tensor network (TTN) approach. This allows us to directly compute the quench dynamics of the transverse-field Ising model (TFIM) on an open 2D square lattice. Because the TTN architecture naturally mirrors 2D lattice connectivity, we can extract the global Loschmidt echo. Our simulations reveal that while deep quenches yield standard DQPTs, quenching within the ferromagnetic phase produces an anomalous dynamical response. In this regime, the rate function exhibits sharp non-analytic peaks even as the macroscopic order parameter maintains its initial sign. This decoupled behavior strongly indicates that local spin excitations drive 2D DQPTs, rather than the macroscopic domain-wall motions seen in 1D chains. These results provide a quantitative numerical baseline for understanding non-equilibrium quantum matter in higher dimensions.

## 1. Introduction

Exploring the real-time evolution of isolated quantum many-body systems far from equilibrium is a difficult task. Dynamic quantum phase transitions (DQPTs) provide a unique theoretical perspective. It is extremely rare for complex many-body systems to exactly return to their initial state after a long period of evolution [1]. We can think of a DQPT as a critical point in the time domain. Until this critical moment is reached, the system retains the memory of its initial state; once this point in time is crossed, quantum fluctuations will dominate the system, leading to singularities in macroscopic properties [1]. Beyond isolated quantum systems, understanding the role of fluctuations and environmental noise remains a central pursuit within the broader framework of statistical physics and complex systems [2].

Unlike conventional phase transitions, which rely heavily on spatial dimensionality and are confined to thermal equilibrium [3,4], DQPTs push the concept of criticality into the realm of real-time dynamics, intertwining with topology, quantum information, and symmetry breaking [5,6,7,8,9,10,11,12,13,14,15,16]. These transitions are typically characterized by two distinct signatures [7]: DQPT-I, involving the macroscopic order parameter oscillating across zero, and DQPT-II, manifesting as non-analytic peaks in the Loschmidt rate function λ(t) exactly when the time-evolved state becomes orthogonal to the initial state. In conventional one-dimensional (1D) systems, these two signatures are strictly synchronized due to the unhindered motion of point-like domain walls. While matrix product states (MPS) have been successfully employed to study these transitions in 1D chains and quasi-1D cylinders [17,18,19,20,21,22,23,24,25,26], the simulation of genuine 2D dynamics remains computationally intensive. Consequently, our understanding of such non-equilibrium phenomena in higher-dimensional systems is still quite limited.

Experimentalists have recently made great strides in directly observing DQPTs using platforms like trapped ions [27,28], ultracold atoms [29,30], and superconducting qubits [31,32,33]. On the numerical front, techniques such as exact diagonalization (ED) and one-dimensional (1D) matrix product states (MPS) are standard tools for calculating real-time evolution [34]. For the standard 1D transverse-field Ising chain (TFIC), the conditions for a DQPT are quite strict. A quantum quench has to cross the quantum critical point (QCP) to trigger the transition. Because 1D point-like domain walls move freely without any extra energy penalty, the DQPT-I and DQPT-II are locked together and happen simultaneously [5,35]. Domain walls in 2D form closed loops, and making these loops larger costs extensive energy—known as string tension. This energy penalty heavily restricts macroscopic domain-wall motion [36].

Past numerical efforts mapped 2D models onto quasi-1D infinite cylinders via MPS. These studies reported anomalous intra-phase DQPTs [36]. Meanwhile, ED approaches are strictly limited to small clusters (like 4×4) due to the exponential growth of the Hilbert space, meaning true thermodynamic signatures remain out of reach [37,38]. To circumvent these quasi-1D cylinder mappings and finite-size bottlenecks, we simulated the real-time quench dynamics directly on an open square lattice using a tree tensor network (TTN) approach [39,40,41,42,43,44,45,46,47]. The TTN’s hierarchical entanglement structure maps naturally onto a 2D grid, keeping unphysical long-range folds to a minimum. The loop-free nature of the TTN allows us compute the global Loschmidt echo exactly. This is a massive computational advantage over projected entangled-pair states (PEPS), where evaluating global wavefunction overlaps is generally #P-hard [48].

Our data support the idea that anomalous DQPTs can appear when quenching entirely inside the ferromagnetic phase. What stands out most is the complete decoupling between the dynamical singularities and the macroscopic order parameter. Specifically, the rate function exhibits sharp non-analytic peaks even though the macroscopic magnetization never crosses zero. Seeing this decoupling tells us that 2D DQPTs are driven by localized spin excitations, not the macroscopic domain-wall motions we see in 1D. Ultimately, we hope this work provides a solid numerical baseline for studying higher-dimensional non-equilibrium dynamics. This paper is structured as follows. Section 2 introduces the 2D TFIM and our TDVP-based TTN algorithm. In Section 3, we show the numerical results, covering both the equilibrium phase diagram and the anomalous quench dynamics. Finally, Section 4 summarizes our findings and points out a few future research directions.

## 2. Theoretical Framework and Numerical Methods

### 2.1. 2D Transverse-Field Ising Model and Observables

We consider the transverse-field Ising model (TFIM) defined on an $N=L_{x}\times L_{y}$ square lattice, governed by the Hamiltonian:

(1) $$H=-J\sum_{\langle i,j\rangle }\sigma_{i}^{z}\sigma_{j}^{z}-h\sum_{i}\sigma_{i}^{x},$$

where J>0 is the ferromagnetic coupling, *h* is the transverse field, and $\sigma^{\alpha }$ (α=x,y,z) are the Pauli matrices. In the thermodynamic limit, the 2D TFIM has a quantum critical point (QCP) at $h_{c}\approx 3.044\;J$ [49,50,51,52,53]. We impose open boundary conditions (OBCs) to simulate a system that is sufficiently large in both spatial directions. Following a sudden quench from an initial state $\psi_{i}$, the dynamics of DQPT-II are captured by the global Loschmidt echo $L\left(t\right)=|\langle \psi_{i}|\psi \left(t\right)\rangle {|}^{2}$. To identify dynamical singularities, we study the rate function λ(t):

(2) $$\lambda \left(t\right)=-\lim_{N\to \infty }\frac{1}{N}\ln L\left(t\right).$$

To establish an analytical baseline, Appendix D provides the exact Loschmidt return rate for the 1D TFIC. This exact solution proves that in 1D systems, a DQPT must strictly cross the equilibrium QCP, and the non-analytic cusps in λ(t) must perfectly synchronize with the zero-crossings of the macroscopic magnetization. In Section 3, we demonstrate how 2D lattice geometry and string tension fundamentally break these 1D constraints.

In our quench protocols, $h_{i}$ denotes the transverse field of the initial Hamiltonian $H(h_{i})$, used to prepare the starting ground state $|\psi_{i}\rangle$, while $h_{f}$ denotes the field strength of the post-quench Hamiltonian $H(h_{f})$. Compared to conventional MPS mappings, the TTN architecture naturally reflects the 2D lattice connectivity through its hierarchical tree structure. Importantly, the loop-free geometry of TTN allows for the efficient evaluation of the global Loschmidt echo. This provides a significant advantage over Projected Entangled-Pair States (PEPS), where contraction is generally #P-hard. However, we note that the tree structure imposes a specific entanglement scaling that may introduce truncation artifacts for highly entangled 2D states, though our convergence tests confirm its reliability for the time scales studied here.

### 2.2. Tree Tensor Network and Evolution Scheme

We sidestep the geometric constraints mentioned earlier by adopting a 2D tree tensor network (TTN) framework [39,54]. This approach offers a two-fold advantage: compared to standard one-dimensional matrix product states (MPS), TTN maps the actual 2D lattice connectivity much more naturally; meanwhile, unlike projected entangled-pair states (PEPS) [48], the loop-free geometry of TTNs allows for highly efficient and exact tensor contractions. We carried out all TTN simulations and real-time evolutions in this study using the open-source Julia library TTN.jl [55].

Our numerical procedure starts by finding the ground state of the initial Hamiltonian. We then take this ground-state wavefunction and evolve it in real time under a post-quench Hamiltonian. For this dynamical step, we rely on the time-dependent variational principle (TDVP) [40,56,57]. Since quantum quenches naturally trigger rapid entanglement growth [34], we handle this by encoding the target maximum bond dimension χ into the state right from the beginning. Thanks to the aforementioned loop-free geometry, we avoid the contraction bottlenecks typical of generic 2D tensor networks, enabling us to evaluate the global Loschmidt echo in Equation (2) both exactly and efficiently.

## 3. Results

### 3.1. Equilibrium Ground-State Phase Diagram and Finite-Size Scaling

Our simulations are performed at absolute zero temperature (T=0 K), where the physics is entirely driven by quantum fluctuations. In the context of the phase diagram, the system operates strictly within the quantum critical region. This ensures that the observed dynamical singularities originate from pure quantum critical dynamics rather than thermal transitions.

**Figure 1:**
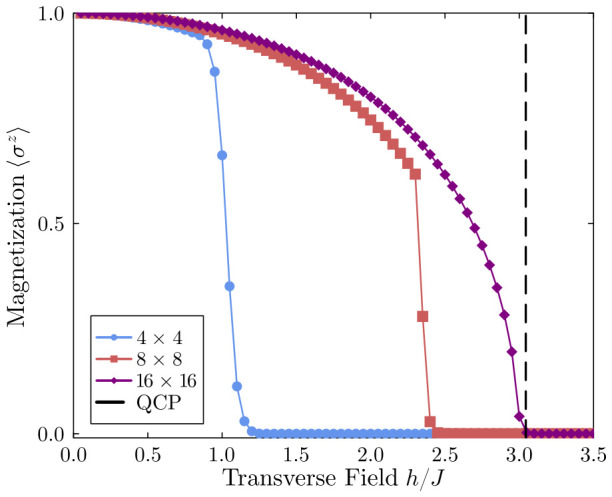
Equilibrium ground-state phase diagram of the 2D transverse-field Ising model across different lattice sizes (4×4, 8×8, and 16×16). The curves reveal a strong finite-size scaling trend. By expanding the lattice, the effective quantum critical point (QCP) steadily approaches the exact thermodynamic limit of ${\left(h/J\right)}_{c}\approx 3.044$. This outward shift occurs because larger systems naturally recover the average coordination number that gets suppressed by open boundaries. All ground states were obtained via the TTN algorithm, taking maximum bond dimensions of χ=60, 120, and 240 for the 4×4, 8×8, and 16×16 systems, respectively.

To establish a rigorous foundation for the non-equilibrium dynamics, we first investigate the equilibrium ground-state properties of the 2D TFIM. Figure 1 illustrates the spontaneous macroscopic magnetization $\langle \sigma^{z}\rangle$ as a function of the scaled transverse field h/J for different lattice sizes. A clear finite-size scaling behavior emerges. In the strict thermodynamic limit (N→∞), the quantum critical point is ${\left(h/J\right)}_{c}\approx 3.044$ [49,50]. Using an open boundary essentially reduces the number of nearest neighbors for spins at the edges and corners. The decrease in the effective coordination number ($z_{\mathrm{eff}}$) makes the global ferromagnetic state highly susceptible to quantum fluctuations. This results in a lower effective QCP in small systems, dropping to $h_{c}^{\mathrm{eff}}\approx 1.1\;J$ in our 4×4 simulation. This boundary effect is gradually corrected by increasing the lattice size, as the bulk spins begin to dominate. The numerical results clearly demonstrate this recovery. We see a further increase in the effective QCP to 2.55J (for the 8×8 lattice), nearing 3.0J (for the 16×16 system). The proximity of the 16×16 data to the thermodynamic limit confirms that the TTN algorithm captures the genuine 2D critical physics.

### 3.2. Non-Equilibrium Quench Dynamics in 8 × 8 Lattices

To observe genuine 2D dynamical features, we use an 8×8 square lattice. This system size is large enough to suppress small-cluster artifacts, yet it still allows our TTN/TDVP real-time evolution to run efficiently. As shown in the previous scaling analysis, the effective critical point for this particular grid is roughly $h_{c}^{\mathrm{eff}}\approx 2.55\;J$. Following the rigorous convergence tests detailed in Appendix B, we utilized a maximum bond dimension of χ=80 alongside a time step of δt=0.01/J for all the 8×8 simulations.

**Figure 2:**
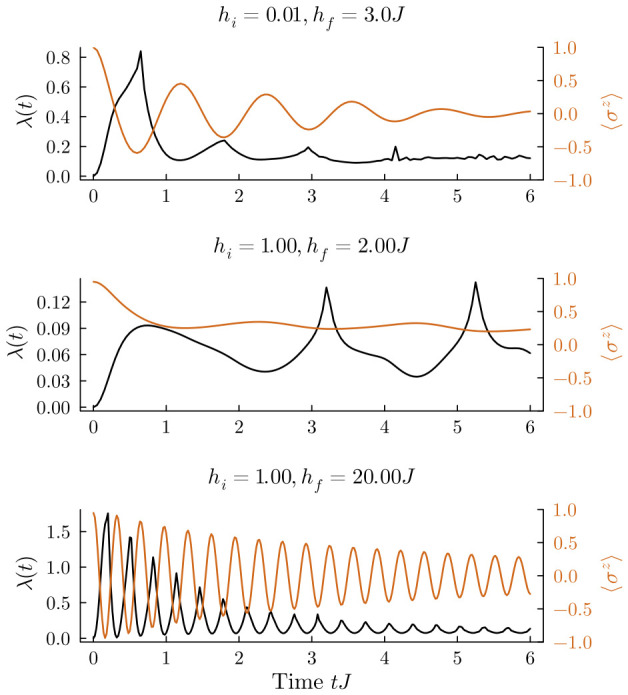
Dynamical quantum phase transitions in the 8×8 transverse-field Ising model. The panels plot the time evolution of the rate function λ(t) (left axis, black lines) alongside the macroscopic magnetization $\langle \sigma^{z}\rangle$ (right axis, orange lines) for three quench setups. **Top panel** (0.01J→3.0J): Quenching across the effective QCP yields a regular DQPT. **Middle panel** (1.00J→2.00J): Quenching within the ferromagnetic phase produces an anomalous DQPT. The order parameter and rate function decouple completely in this regime: λ(t) develops non-analytic peaks, but $\langle \sigma^{z}\rangle$ remains strictly positive without any sign reversal. This confirms that local spin flips are driving the transition. **Bottom panel** (1.00J→20.00J): Under a large-amplitude quench, the 2D lattice behaves much like the 1D TFIM. We see periodic sign reversals in the order parameter synchronizing perfectly with the singularities in the rate function.

Figure 2 systematically outlines the non-equilibrium responses under three distinct quench setups. The top panel ($h_{i}=0.01\;J\to h_{f}=3.0\;J$) depicts a quench crossing the effective phase boundary. We see a regular DQPT here: the macroscopic magnetization decays rapidly and crosses zero as the system is thrust into the paramagnetic phase. The middle panel ($h_{i}=1.00\;J\to h_{f}=2.00\;J$), however, shows an anomalous DQPT caused by quenching strictly inside the ferromagnetic region. The rate function λ(t) still produces non-analytic singularities, but the macroscopic magnetization never changes sign. We can attribute this effect to the interface roughening transition recently identified within the 2D ferromagnetic phase [58,59,60,61,62,63,64,65]. When quenched into this regime, smooth domain walls are unstable. Instead, the dynamics are governed by localized spin flips. Because these local excitations can independently drive singularities in the rate function, the system bypasses the need for global domain-wall inversions. This behavior departs sharply from the conventional paradigm established in 1D models.

In the large-amplitude quench regime ($h_{i}=1.00\;J\to h_{f}=20.00\;J$, bottom panel of Figure 2), the 2D system recovers a 1D-like synchronization. The periodic inversion of the order parameter align perfectly with the non-analytic peaks in λ(t). This occurs because the massive energy injection overcomes the string tension, allowing for rapid, macroscopic domain-wall inversions similar to those seen in 1D chains.

### 3.3. Microscopic Mechanism of the Anomalous DQPT

**Figure 3:**
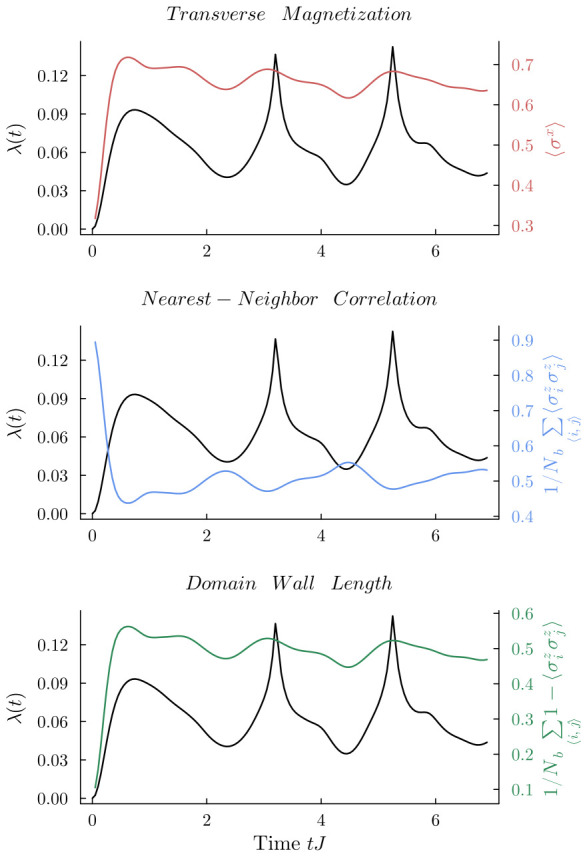
Dynamical evolution of the rate function λ(t) (**top**), transverse magnetization $\langle \sigma^{x}\rangle$ (**middle**), and domain-wall length $1/N_{b}\sum_{\langle i,j\rangle }\left(1-\langle \sigma_{i}^{z}\sigma_{j}^{z}\rangle \right)$ (**bottom**) for the anomalous quench $h_{i}=1.00\;J\to h_{f}=2.00\;J$ on an 8×8 lattice. The local maxima of both the transverse magnetization and the domain-wall length perfectly synchronize with the non-analytic peaks of λ(t). This strict temporal alignment visually confirms that the anomalous dynamical phase transition is driven by a sudden proliferation of localized spin–flip excitations, which is fundamentally distinct from the global macroscopic domain inversions seen in conventional cases.

To understand the microscopic origin of the anomalous DQPT, we study the $h_{i}=1.00\;J\to h_{f}=2.00\;J$ quench, as shown in Figure 3. In this case, the macroscopic magnetization $\langle \sigma^{z}\rangle$ does not invert throughout the evolution. However, the rate function λ(t) still exhibits sharp non-analytic peaks.

To explain this phenomenon, we calculate the transverse magnetization $\langle \sigma^{x}\rangle$ and the domain wall length. The domain wall length is defined as $1/N_{b}\sum_{\langle i,j\rangle }\left(1-\langle \sigma_{i}^{z}\sigma_{j}^{z}\rangle \right)$, where $N_{b}$ is the total number of nearest-neighbor bonds. This quantity directly measures the number of anti-parallel spin pairs, representing local excitations. As illustrated in Figure 3, the peaks of λ(t) perfectly synchronize with the local maxima of $\langle \sigma^{x}\rangle$ and the domain wall length. This demonstrates that at the critical times, the system generates a high density of localized spin-flip excitations.

Notably, the curve of $\langle \sigma^{x}\rangle$ has the exact same shape as the domain wall length. This is due to energy conservation in our closed system. Since the total energy is constant, the transverse field energy (related to $\langle \sigma^{x}\rangle$) must be strictly proportional to the interaction energy (related to the domain wall length).

## 4. Discussion and Conclusions

We have utilized a tree tensor network (TTN) geometry to compute both the equilibrium phase diagram and the real-time quench dynamics of the 2D transverse-field Ising model. By increasing the lattice size, we successfully tracked the 2D quantum critical point. The TTN framework proves especially useful here: it preserves the 2D spatial connectivity without the prohibitive computational costs of PEPS. Because TTNs are loop-free, evaluating the exact global Loschmidt echo becomes a straightforward and efficient operation.

Two-dimensional non-equilibrium dynamics deviate sharply from one-dimensional expectations. A prime example is the anomalous dynamical phase we found for quenches strictly inside the ferromagnetic region. In this specific case, the Loschmidt echo rate function develops clear non-analytic peaks, even as the macroscopic magnetization avoids crossing zero entirely. This temporal decoupling strongly suggests that macroscopic domain-wall inversions do not drive 2D DQPTs. Instead, localized spin-flip excitations triggered by interface instabilities are responsible for the singularities. To test this microscopic picture further, future work should track the dynamics of nearest-neighbor spin correlations. Ultimately, we hope this study clarifies the true nature of 2D DQPTs and sets a practical benchmark for modeling non-equilibrium quantum matter.

Furthermore, while our current TTN framework assumes strictly unitary evolution, real experimental platforms are inevitably subject to environmental noise. As highlighted by fundamental studies in complex systems, mesoscopic transport, and thermalization [66,67,68,69,70,71,72,73,74,75,76,77], investigating how stochastic fluctuations influence the robustness of these 2D DQPTs represents an important direction for future research.

## Figures and Tables

**Figure A1:**
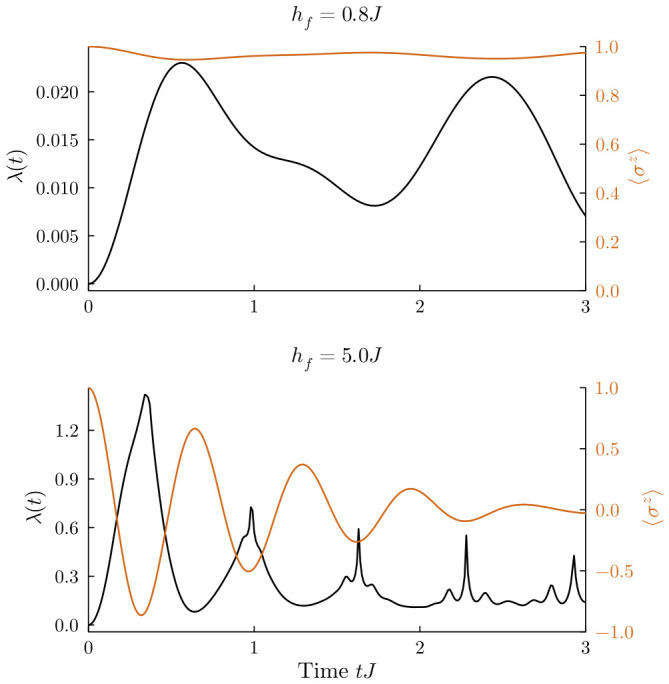
Dynamical evolution of the rate function λ(t) (left axis, black lines) and macroscopic magnetization $\langle \sigma^{z}\rangle$ (right axis, orange lines) for a 4×4 system initialized in the ferromagnetic phase. **Top panel** ($h_{f}=0.8\;J$): A shallow quench yields smooth analytic evolution without DQPTs due to the large finite-size gap. **Bottom panel** ($h_{f}=5.0\;J$): A deep quench triggering a regular DQPT where the non-analytic peaks in λ(t) synchronize perfectly with the sign reversals of $\langle \sigma^{z}\rangle$. Notably, the anomalous decoupled DQPT observed in larger systems is entirely absent here, highlighting that the anomalous phase intrinsically requires extended 2D spatial correlations.

**Figure A2:**
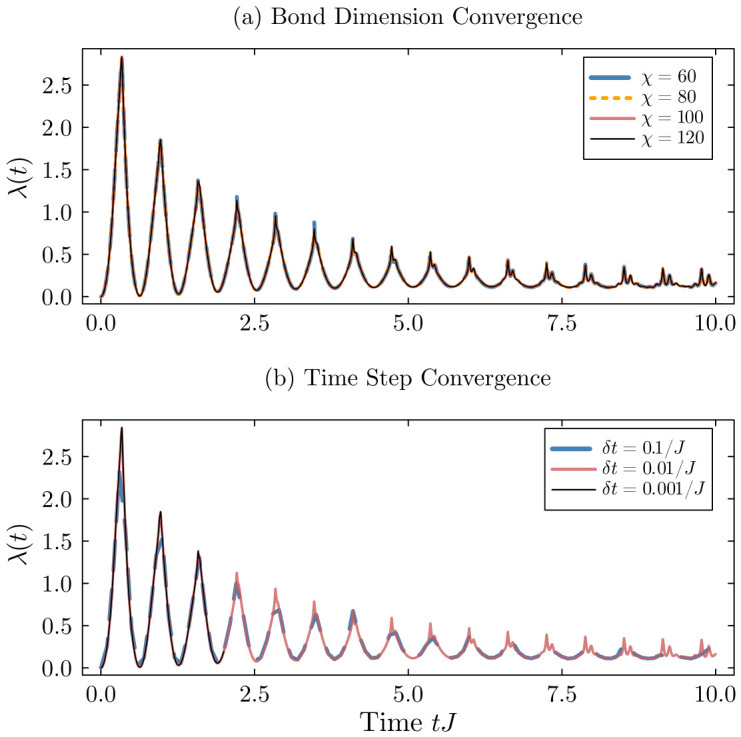
Finite-entanglement scaling and convergence test of the TTN evolution for a deep quench (0.01J→10.0J). The temporal trajectories show exact numerical collapse for maximum bond dimensions χ=60 and χ=120. This perfect agreement up to t=10.0/J rigorously confirms that the observed dynamical singularities are robust physical phenomena, free from truncation-induced artifacts.

**Figure A3:**
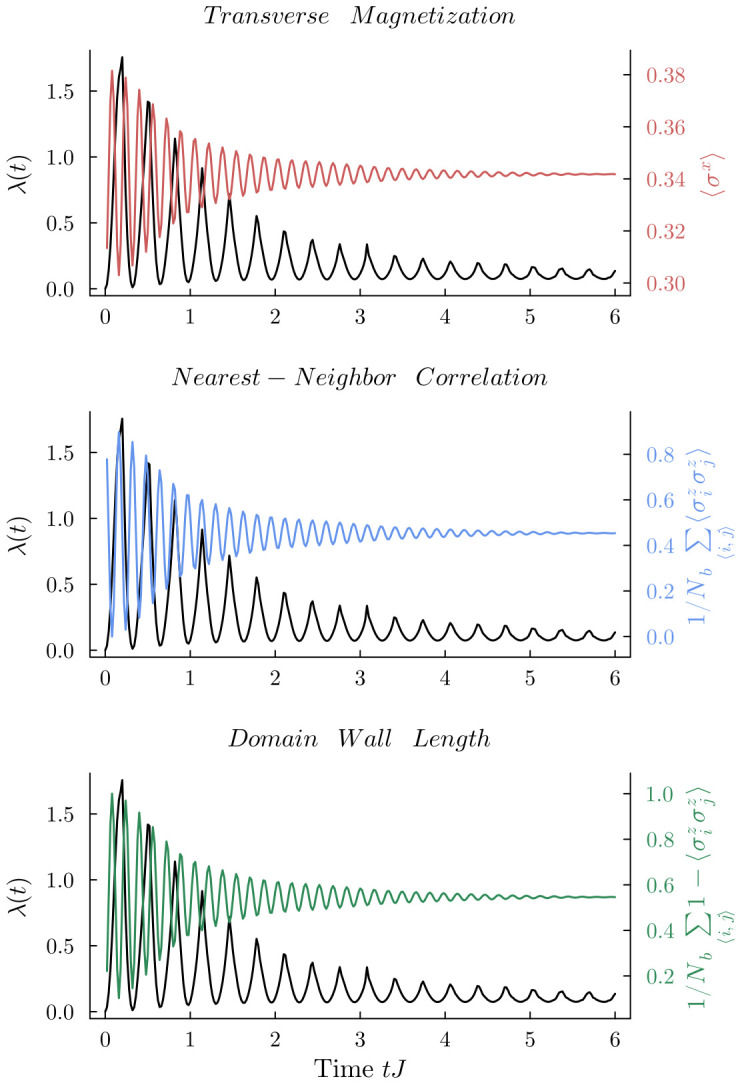
Dynamics of the conventional DQPT under a deep quench $h_{i}=1.00\;J\to h_{f}=20.00\;J$. The panels compare the rate function λ(t) with the transverse magnetization $\langle \sigma^{x}\rangle$ (**top**), nearest-neighbor correlation $\langle \sigma_{i}^{z}\sigma_{j}^{z}\rangle$ (**middle**), and domain wall length (**bottom**). In stark contrast to the anomalous case, the non-analytic peaks of λ(t) coincide exactly with the local minima of the domain wall length (approaching zero). This indicates the near absence of spatial domain walls at the critical times, confirming that this conventional transition is driven by a collective, global spin inversion into a fully reversed ferromagnetic state.

## Data Availability

Data is contained within the article.

## Appendix A. Finite-Size Effects and Quench Dynamics in 4 × 4 Lattices

To highlight the necessity of simulating sufficiently large lattices, Figure A1 illustrates the time evolution of a small 4×4 system initialized deep in the ferromagnetic phase. For a shallow quench strictly within the effective ferromagnetic phase (e.g., $h_{f}=0.8\;J$), the injected energy cannot overcome the large finite-size gap of the small cluster. Consequently, the magnetization remains robustly polarized, and the rate function λ(t) undergoes only smooth, analytic oscillations without any dynamical phase transitions. Conversely, a deep quench into the paramagnetic phase ($h_{f}=5.0\;J$) overwhelmingly overcomes this finite-size gap. In this regime, the magnetization rapidly decays and exhibits zero-crossing oscillations, strictly synchronized with the emergence of non-analytic peaks in λ(t), signifying a regular DQPT. Crucially, the anomalous dynamical phase observed in the 8×8 system—where the non-analytic peaks completely decouple from the magnetization zero-crossings—is entirely absent in this 4×4 lattice. This stark contrast demonstrates that the anomalous DQPT is a genuine higher-dimensional phenomenon requiring a sufficiently large spatial correlation length. In small clusters, geometric truncation severely restricts spatial degrees of freedom, effectively suppressing the localized spin excitations necessary to drive the anomalous decoupled phase.

## Appendix B. Convergence of the Tree Tensor Network Evolution

To rigorously rule out spurious singularities caused by finite entanglement truncation and time discretization, we perform systematic convergence tests over the maximum bond dimension χ and the time step δt. As demonstrated in Figure A2 for the quench protocol 0.01J→10.0J, the real-time trajectories of both the rate function λ(t) and macroscopic magnetization $\langle \sigma^{z}\rangle$ perfectly converge for bond dimensions χ=60,80,100, and 120, as well as for time steps δt=0.01/J and 0.001/J. Taking computational efficiency into account without compromising numerical accuracy, all 8×8 dynamical simulations presented in the main text are consistently performed using a maximum bond dimension of χ=80 and a time step of δt=0.01/J. This confirms that our observed dynamical singularities are robust physical phenomena, completely free from numerical artifacts.

## Appendix C. Comparison with Conventional DQPTs

In this appendix, we analyze conventional DQPTs for comparison, focusing on a deep quench (1.00J→20.00J).

For the 1.00J→20.00J quench in Figure A3, the behavior is the opposite of the anomalous case. When λ(t) reaches its peaks, the domain-wall length drops to its local minima. A minimum domain-wall length means that almost all local spins are parallel, indicating the absence of domain walls in the space. At these exact times, the macroscopic magnetization $\langle \sigma^{z}\rangle$ reaches a completely reversed sign (a negative peak). This confirms that this highly parallel state is actually a fully reversed ferromagnetic state. Thus, conventional DQPTs in deep quenches are driven by a collective, global spin inversion rather than the proliferation of localized domain walls.

## Appendix D. Exact Solution and DQPTs in the 1D Transverse-Field Ising Chain

This appendix briefly outlines the exact analytical conditions for dynamical quantum phase transitions (DQPTs) in the one-dimensional nearest-neighbor transverse-field Ising chain (TFIC). The Hamiltonian is given by:

(A1) $$H=-\sum_{i}\left[J\sigma_{i}^{z}\sigma_{i+1}^{z}+h\sigma_{i}^{x}\right].$$

By mapping the spin operators to spinless free fermions via the standard Jordan-Wigner, Fourier, and Bogoliubov transformations [78,79], the intensive logarithmic Loschmidt return rate in the thermodynamic limit can be exactly derived as [5]:

(A2) $$\lambda \left(t\right)=-\int_{-\pi }^{\pi }\frac{dk}{2\pi }\ln\left[1-\sin^{2}\left(\theta_{k}^{f}-\theta_{k}^{i}\right)\sin^{2}\left(2\epsilon_{k}^{f}t\right)\right],$$

where $\epsilon_{k}^{f}$ is the post-quench quasiparticle dispersion, and $\theta_{k}$ is the Bogoliubov angle. For λ(t) to exhibit non-analytic cusps (i.e., to trigger a DQPT), the argument of the logarithm must vanish. The spatial condition requires the interference amplitude to be maximal ($\sin^{2}\left(\theta_{k}^{f}-\theta_{k}^{i}\right)=1$). This yields the critical momentum $k_{c}$ [36]:

(A3) $$\cos k_{c}=\frac{J^{2}+h_{i}h_{f}}{J(h_{i}+h_{f})}.$$

For $k_{c}$ to be a valid real solution, we must have $\cos k_{c}\leq 1$. Assuming $J,h_{i},h_{f}>0$, rearranging this inequality gives:

(A4) $$\left(J-h_{i}\right)\left(J-h_{f}\right)\leq 0.$$

This mathematical constraint rigorously proves that $h_{i}$ and $h_{f}$ must lie on opposite sides of the equilibrium quantum critical point ($h_{c}=J$). In other words, a 1D DQPT can onlyoccur if the quench strictly crosses the phase boundary. Furthermore, these critical times strictly synchronize with the zero-crossings of the macroscopic order parameter. Therefore, the 1D TFIC exclusively hosts regular DQPTs, fundamentally distinguishing it from the anomalous intra-phase decoupling observed in genuine 2D lattices.
